# Achieving stable myocardial regeneration after apical resection in neonatal mice

**DOI:** 10.1111/jcmm.15223

**Published:** 2020-04-28

**Authors:** Yandong Li, Jie Feng, Yan Li, Shengshou Hu, Yu Nie

**Affiliations:** ^1^ State Key Laboratory of Cardiovascular Disease Fuwai Hospital National Center for Cardiovascular Disease Chinese Academy of Medical Sciences and Peking Union Medical College Beijing China; ^2^ Department of Biochemistry and Molecular Biology Shanxi Medical University Taiyuan China

**Keywords:** apical resection, heart regeneration, whole‐heart‐slice

## Abstract

The neonatal heart completely regenerates after apical resection (AR), providing a desirable research model to study the mechanism of cardiac regeneration and cardiomyocyte proliferation. However, AR‐induced neonatal heart regenerative phenomenon is controversial due to the variation of operative details in different laboratories. Here, we provide an optimized AR operation procedure with stable regeneration and high survival rate by achieving heart exposure, normalizing myocardium cut‐offs, and reducing operation duration. We also established a whole‐heart‐slice approach to estimate the myocardial regeneration after the AR operation, which ensures no false‐negative/positive results. The combination of the optimized AR operation and the whole‐heart‐slice analysis provides a stable system to study neonatal heart regeneration and cardiomyocyte proliferation *in situ*.

## 1 INTRODUCTION

Heart failure is a refractory disease involving a heavy loss of cardiomyocytes and a poor myocardial regeneration capacity in adults after cardiac injury.^1^ Turning regenerative technologies into cardiac repair treatments after myocardial injuries is a promising strategy to treat heart failure.^1^ It is of difference from adults that neonatal mouse hearts can completely regenerate at 21 days after apical resection (AR) with cardiomyocyte proliferation,^2^ providing an ideal mammalian model to investigate the mechanism of mammalian heart regeneration and explore relevant therapeutic targets.^3^, ^4^, ^5^

With the popularization of neonatal mouse AR model in heart regeneration study, simultaneously, the controversies of AR‐induced neonatal heart regenerative phenomenon are continuous.^6^, ^7^, ^8^, ^9^ Alternatively, false‐negative/positive results could also be introduced when inaccurate analytical layers are enrolled in the pathological analysis to evaluate myocardial regeneration, even in the context of a unified AR operation.^10^ It is essential to establish an easy standardized AR operation and a reliable myocardial regeneration estimation system, which is beneficial for researchers pursued in heart regeneration study.

In this study, we provide a detailed protocol of an optimized AR operation with exteriorization of the heart for apex cutting, which makes it easy to unify the amputation size under visual inspection. To avoid false judgment of heart regeneration, we employ a whole‐heart‐slice approach to evaluate the myocardium growth after AR operation. With the optimized AR operation and whole‐heart‐slice approach, researchers can obtain a stable regenerative phenomenon after neonatal cardiac injury.

## 2 MATERIALS AND METHODS

### 2.1 Mice

Neonatal 1‐day‐old mice of both sexes were used in our study. The *C57BL/6J wildtype (WT)* mice were obtained from Vital River Laboratory Animal Technology Co. Ltd. The *IL‐6 knockout (KO)* (B6.129S2‐Il6tm1Kopf/J) mice were acquired from the Jackson Laboratory. All experiments involving animals were conducted in accordance with the Guide for the Use and Care of Laboratory Animals. All animal protocols were approved by the Institutional Animal Care and Use Committee (IACUC), Fuwai Hospital, Chinese Academy of Medical Sciences.

### 2.2 Histological analyses

Mice were killed at 21 days post‐resection (dpr), and the hearts were gently harvested without any other extraction. Dissected hearts were rinsed in sterile PBS until they stopped beating and then fixed in 4% paraformaldehyde at 4°C for 48 hours. Hearts were dehydrated automatically in ethanol and dimethyl benzene after washing by fluent water. Dehydrated heart tissue was embedded in paraffin and sectioned. Whole heart slice was applied afterwards, and each section was mounted on a separate glass slide for further pathological staining.

Masson's trichrome staining was performed on heart section slides following manufacturer's instructions (Sigma‐Aldrich). Images were captured using the Automatic glass scanning system (Zeiss) and were identified myocardium (red) and fibrotic tissue (blue) by at least two researchers. GraphPad Prism software (6.0) was used for statistical analysis and Student's *t* test was used to show the differences.

### 2.3 Statistical analysis

All data were expressed as the mean ± standard error of the mean. Student's unpaired *t* test was used for assessing statistical differences between the two groups, whereas comparisons among more than two groups were performed using the analysis of variance. The results with *P* <.05 were considered statistically significant.

## 3 RESULTS AND DISCUSSION

### 3.1 Exteriorization of the heart enabled to precisely localize the ventricular apex by visual inspection

**FIGURE 1:**
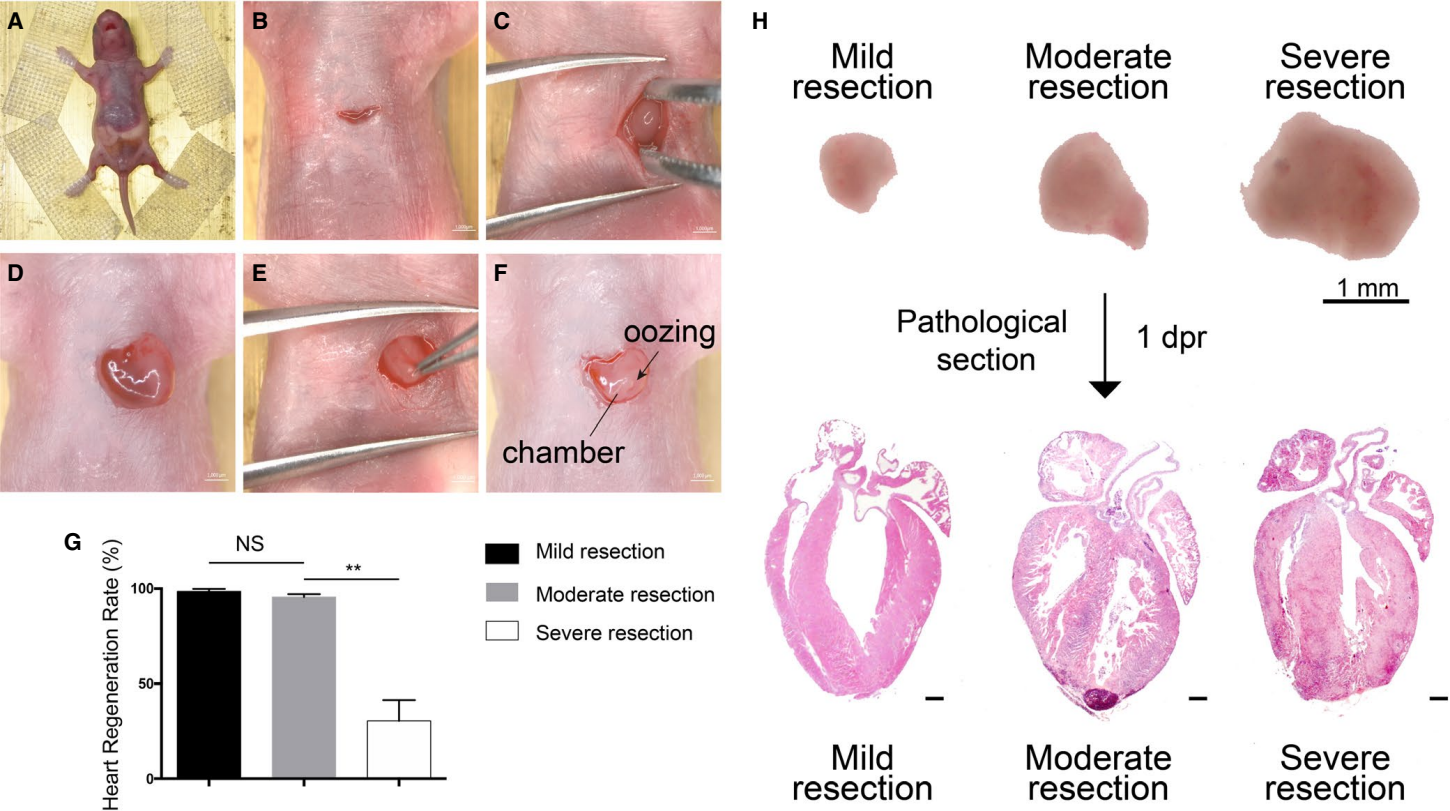
Thoracotomy optimization in neonatal mouse apical resection model. A, Mouse was fixed on the operating table in the supine position. B, The skin was incised for 1 cm long. C, The heart was exteriorized out of the chest cavity with the help of a forceps. D, The heart was immobilized by surrounding chest tissues. E, Clamping the heart out of the chest damaged the heart tissue mechanically. F, The ventricular chamber was exposed with one cut. Exposure of the chamber was the landmark of successful resection. G, Heart regeneration rate of different sizes of resection. n = 20 each. Data are mean ± SEM. NS, no statistical significance, **P < .01. H, Illustration of different cut sizes. Microscopic images (upper) depict HE staining (down). Mild resection (<0.5 mm) was too small to properly expose the chamber. Severe resection (>1.5 mm) could easily expose the chamber but with a lower regeneration rate. Moderate resection (about 1 mm) was the perfect size to replicate the model. 1dpr, 1 day post‐resection

The size of the amputated ventricular apex greatly influences the heart regeneration after AR operation.^11^ Without effective exposure of heart during the AR operation, the size of the resected apex is hard to standardize. To perform an AR operation under better visual inspection, we anchored the mouse on a pre‐cold bronze operating table (Figure 1A) following which the skin and intercostal muscles were incised transversely along the fourth intercostal area of the chest cavity (Figure 1B). We used microsurgical forceps constantly applying pressure alternately on the chest and abdomen; at the same time, ophthalmic forceps were used to set up as a tract to guide the heart out of the chest (Figure 1C). The heart was exteriorized with the guidance of the forceps, which could precisely transmit the pressing force from the operator's hand to the mouse and avoid extra mechanical damage to the heart^11^. A reported AR operating method pulled out the heart using a needle inserted above the resected ventricle to exteriorize,^12^ which could induce extra myocardial damage, protract operation duration and hamper regeneration. In most cases, the neonatal mouse heart can pop out of the chest following our operation. Infrequently, the heart was hard to exteriorize when it was fixed by the pericardium, which could be overcome by tearing the pericardium with forceps.

Approximately, a 1‐cm incision thoracotomy was sufficient for the consequent operation (Figure 1B), including heart exposure and apex resection. The intercostal muscles were separated using scissors until the heart was accessed. The surrounding chest tissues of the small incision served as a natural fixation to immobilize the heart without any injury (Figure 1D), which facilitated the operation on the myocardium. During thoracotomy, it is vital to avoid lung injury when incising the skin transversely along the fourth intercostal area of the chest cavity using microsurgical scissors. A larger incision on the chest is not recommended, such as incising a cut throughout the whole chest,^13^ as it causes more blood loss and prolongs operation time with extra sutures, increasing the risk of death. We repeated an online protocol^6^ of thoracotomy with a transverse skin incision across the chest by using scissors and forceps, causing an injury too large to ensure recovery. When the heart was pulled out of the chest by clamping the apex with forceps, a definitely severe cardiac damage would be induced, rather than apical resection (Figure 1E).

Several researchers do not use stereoscope nor exteriorize the heart out of the chest,^12^, ^13^ which makes it hard to ensure the cutting size with poor operability. A better exposure of the heart is helpful for the localization and then resection of the ventricular apex.^12^ Our optimized AR operation protocol could effectively expose the heart while minimizing the incision for a better operative field, after which a standardized size of the amputated apex is guaranteed.

### 3.2 Standardization of ventricular apex resection size

It has been generally accepted that a cut‐off of 10%‐15% of the heart apex by measuring the weight of the amputated part is proper in the AR model.^14^ However, it is difficult to measure the weight of the cutting part accurately^10^ because of the influence of bleeding during resection. Here, we resorted to the length of the ventricular cutting part instead of weight as a measurement in our operation. We found that cutting off 1 mm in diameter of the resected apex tissue by iridectomy scissors was appropriate when the left ventricular chamber began oozing (Figure 1F), achieving a regeneration rate of >90% (Figure 1G, n = 20). The diameter of the resected cardiac tissue was measured by the electronic ruler attached in the stereoscope under which the operation was performed. A mild resection, which was shorter than 0.5 mm in diameter, might cause false‐positive results. Severe resection, longer than 1.5 mm, might cause death or affect the capacity of myocardial regeneration (regeneration rate <45%, Figure 1G, n = 20; Figure 1H).

Due to the inadequate standardizations of the cutting size, some researchers cannot guarantee a stable regenerative phenomenon^6^, ^7^ and therefore hinder the further popularization and application of the neonatal mouse heart AR model. Our optimization provides a feasible approach to reaching the standard cardiac apex amputation size, which is crucial for the stability of regenerative capacity in the AR model (Table 1).

**TABLE 1:** Technical differences in apical resection operation among laboratories

Reference	Mahmoud et al^10^	Andersen et al^6^	Bryant et al^11^	Xiong and Hou^13^	Notari et al^12^	This study
Mouse strain	ICR/CD‐1	C57BL/6 & ICR/CD‐1	ICR/CD‐1	C57BL/6	ICR/CD‐1	C57BL/6
Foster mother	Yes	No		Yes	Not mention	No
Anaesthesia time	On the ice bed 3‐5 min	On the ice bed 4 min	On the ice bed 4 min	On the ice bed 4 min	On the ice bed 3‐5 min	On the ice bed 2‐3 min
Stereomicroscope	No	Yes	Yes	No	No	Yes
Thoracotomy	Exteriorize the heart outside the chest cavity by applying a steady pressure on the abdomen	A microsurgical forceps was utilized to gently fix the apex	Gently fixed the left ventricle with a microneedle holder	By hand, gently apply pressure on the abdomen to exteriorize the apex of the heart	Gently lifted the left ventricular apex upwards using a needle inserted above the portion to be resected	The heart was exteriorized with the help of forceps
Survival rate	70%	80%‐85%	70%	60%	80%	90%
Regeneration	Yes	No	Yes	Yes	Yes	Yes
Assessment region	Entire ventricular	Whole heart tissue	3‐5 regions throughout the left ventricular chamber	Entire ventricular	Consecutive serial 5‐um‐thick section spanned throughout the injury area of the heart	Whole heart slice

Besides the size of the resected apex tissue, the cutting angle is also an important factor affecting the capacity of regeneration in the AR model. The optimal angle of amputating the apex was 60‐80° to the long axis (LA) of the heart (Figure S1A). The cutting angle should be monitored and standardized by a protractor at the beginning, which would become unnecessary when researchers could skillfully manipulate the operation. An inclination smaller than 60° (Figure S1B) or >80° (Figure S1C) was insufficient to expose the left ventricular chamber even the cutting size was desirable (1 mm) and therefore lead to false‐positive results. Furthermore, if the cutting angle was >90°, researchers would remove the right ventricular tissue undeservedly (Figure S1D), leading to severe cardiac apical resection and suspension of myocardial regeneration.^8^

During the operation, the performer should remove the heart apex by one cut only, which requires a lot of practice to achieve. Superfluous cuts^10^, ^13^ on the heart apex cause excessive heart damage which impairs myocardial regeneration (Figure S2). The whole operation procedure should be finished within 5 minutes.

### 3.3 Assessment of AR‐injured heart regeneration by whole heart slice

Measurement of myocardial regeneration after AR operation is essential to evaluate whether the AR model is successfully established. Previous studies appraise neonatal mouse heart regeneration at 21 days after the AR operation in 1‐day‐old mice by pathological staining with few tissue sections.^11^ In our study, we found that researchers would miss the fibrosis scars induced by AR injury if inadequate regions of heart tissue sections were chosen, which would  lead to an inaccurate evaluation of myocardial regeneration. Our previous study reported that IL‐6 deleyion (*IL‐6 KO*) is harmful for neonatal heart regeneration after AR injury.^15^ However, we found that the fibrosis scars are easily ignored when researchers do not enroll appropriate regions for pathological staining and obtain a false‐negative result (Figure S3).

**FIGURE 2:**
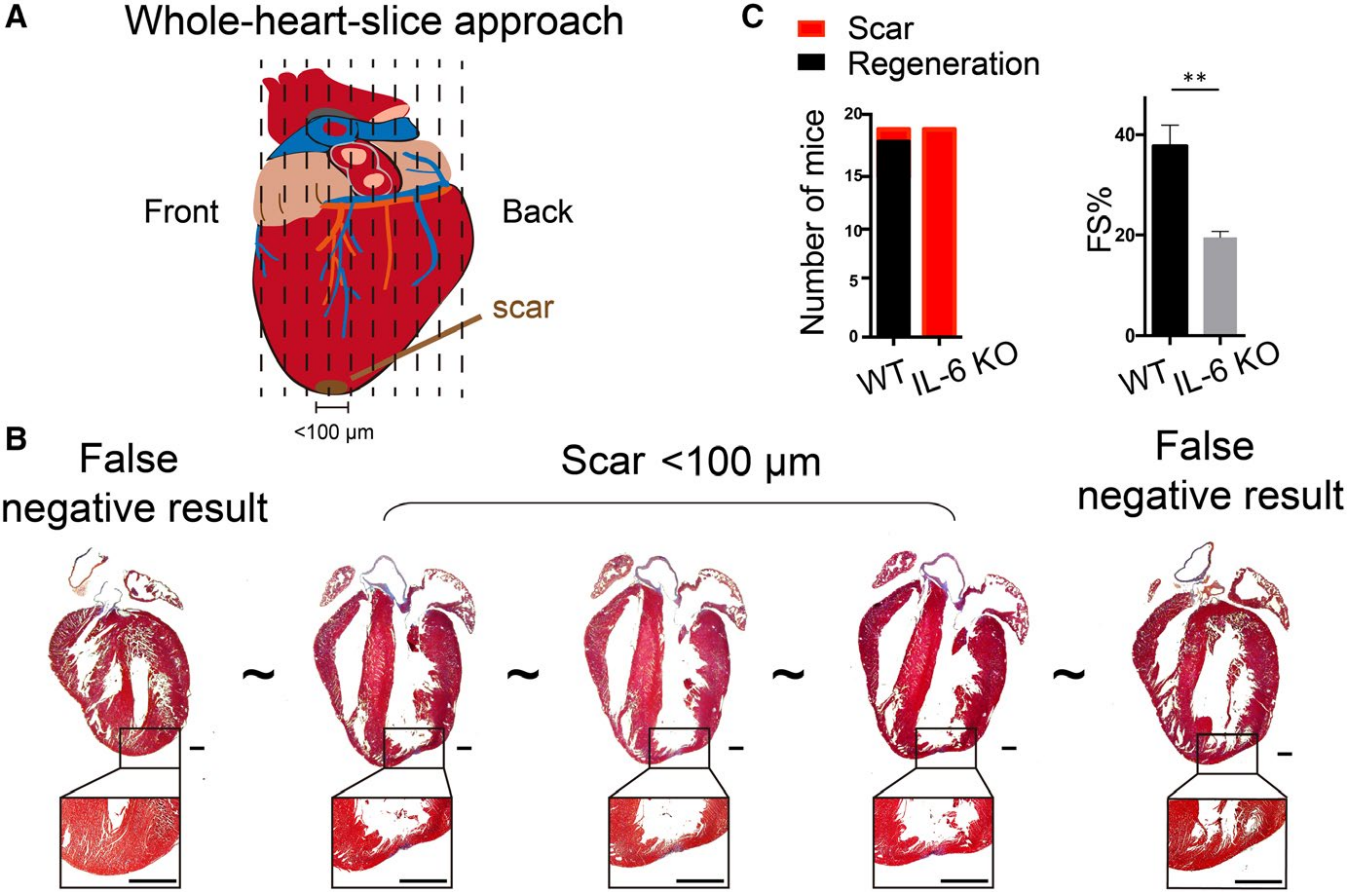
Assessment of myocardial regeneration after apical resection injury in neonatal mice with whole‐heart‐slice approach. A, Schematic of whole‐heart‐slice approach. B, The heart tissue was entirely sliced from front to back. Scale bars are 500 μm. C, Echocardiography data were consistent with the whole‐heart‐slice results

To avoid a false judgement of heart regeneration after AR injury, here we established a whole‐heart‐slice approach (Figure 2A). We sliced the heart tissue longitudinally from the front to the back with a 3‐4 μm each interval and obtained approximately 90 slices totally. We analysed all the sections under the microscope after Masson's staining and found that all the scars could be detected in *IL‐6 KO* mouse hearts at 21 dpr even fibrosis regions were thinner than 100 μm (Figure 2B). Our echocardiography data illustrated that none of the *IL‐6 KO* mouse hearts could recover the pumping function at 21 dpr, which was consistent with the whole‐heart‐slice results (Figure 2C).

## 4 CONCLUSION

We provide a stable and feasible operation for neonatal mouse cardiac AR injury model, which includes exteriorization of the heart, standardization the size of the resected cardiac tissue, and specification of cutting angles. A whole‐heart‐slice approach was established to estimate myocardial regeneration after AR injury, which could avoid false‐negative/positive results. The combined application of the optimized AR operation and the whole‐heart‐slice approach would assist researchers to obtain more stable myocardial regenerative response to AR injury in neonatal mice, which might be helpful to reduce the controversy of AR‐induced neonatal heart regeneration.

## 5 LIMITATIONS

Here, we optimized AR operation on *C57BL/6* mice, which is the most common strain to establish transgenic mice. It is necessary to reappraise the myocardial regenerative ratio when researchers want to perform AR operation following our procedures on the other mouse strains. However, the most procedures we optimized are generally applicable, such as exteriorization of the heart, specification of cutting angles and the whole‐heart‐slice approach.

## CONFLICT OF INTEREST

The authors declare that they have no conflicts of interest with the contents of this article.

## AUTHOR CONTRIBUTIONS

LYD and NY designed the study. LYD collected and analysed the data. FJ and LY performed the histological staining. LYD and FJ drafted and wrote the manuscript. NY and HSS revised the manuscript. All authors read and approved the final manuscript.

## Supporting information

Fig S1

Fig S2

Fig S3

Supplementary Material

## Data Availability

The data that support the findings of this study are available from the corresponding author upon reasonable request.
