# Supplementary figures and images for: Achieving stable myocardial regeneration after apical resection in neonatal mice
Source: J Cell Mol Med. 2020 Apr 28;24(11):6500–4. doi: 10.1111/jcmm.15223 (PMC7294131; doi:10.1111/jcmm.15223)

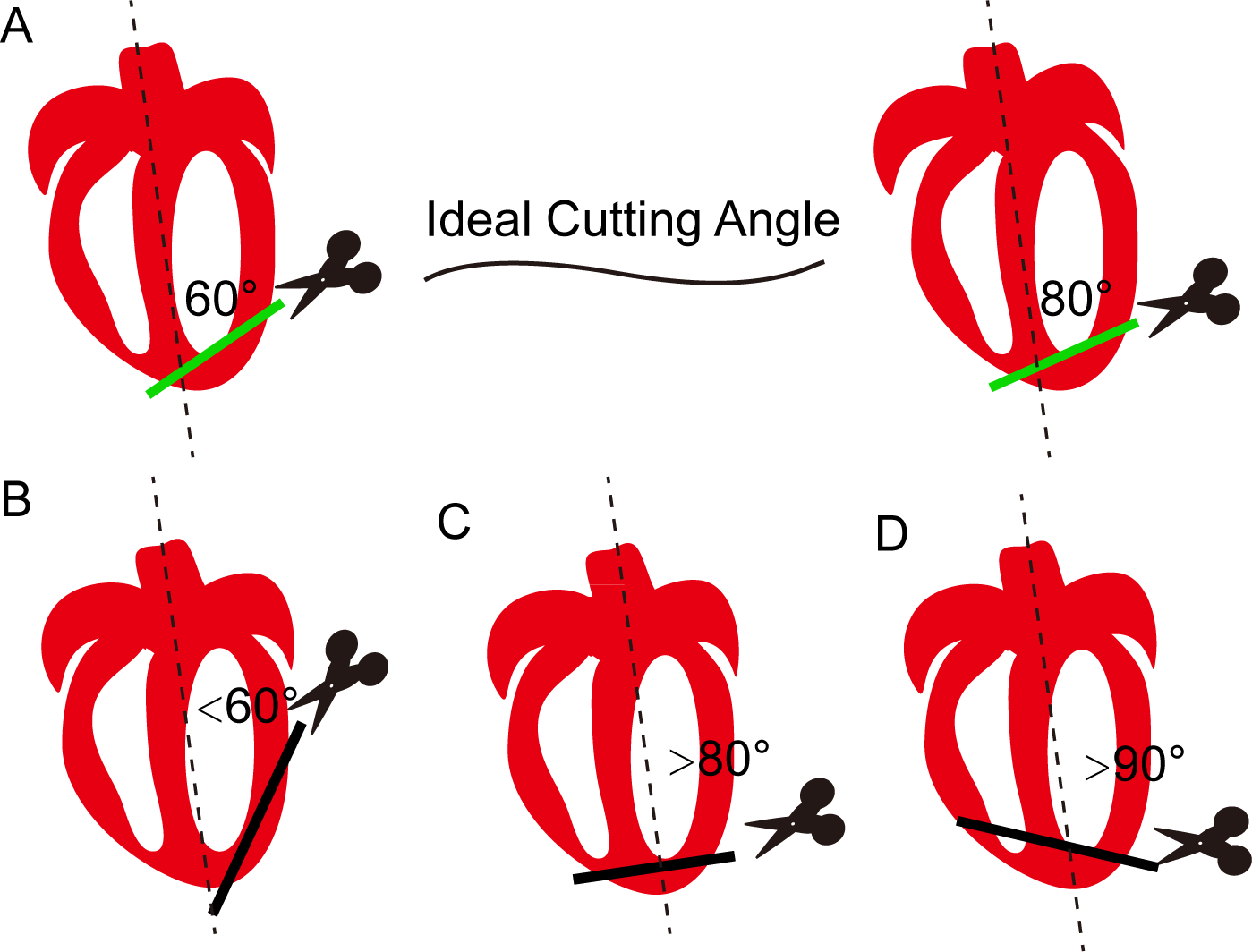

Supplement: Supplementary file 1 — Fig S1 [file JCMM-24-6500-s001.tif]

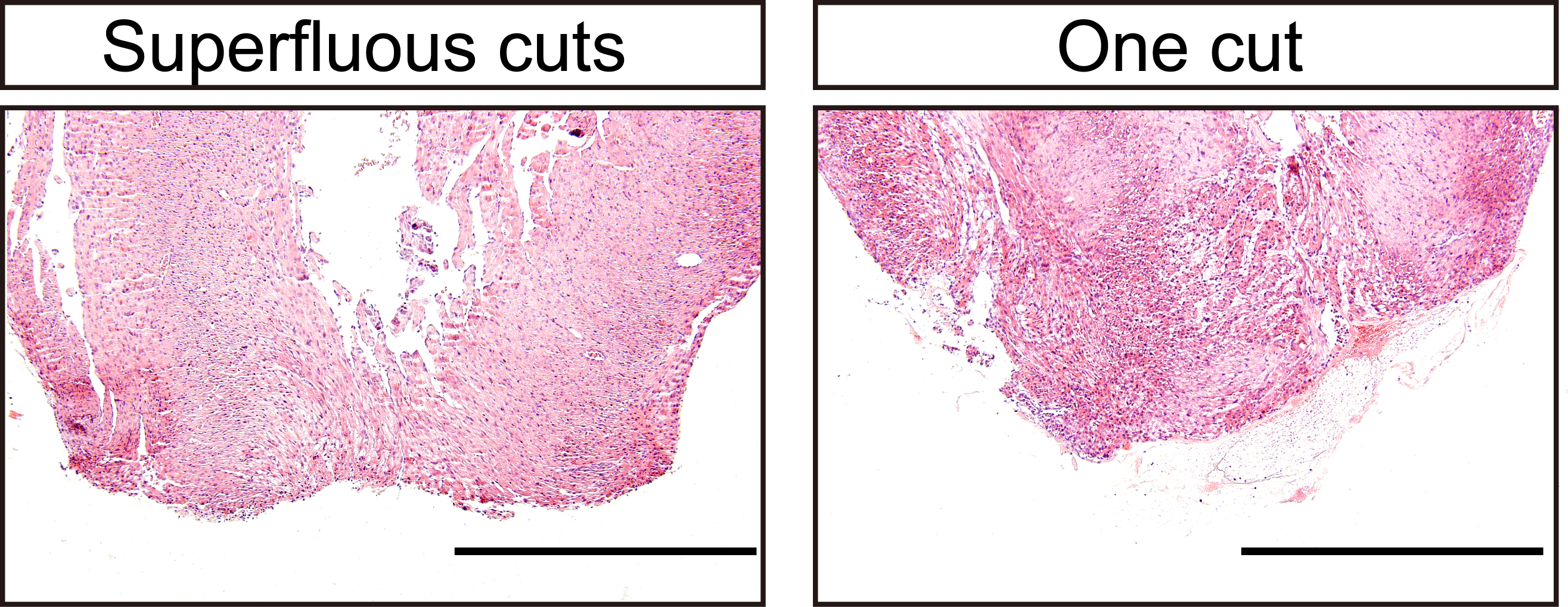

Supplement: Supplementary file 2 — Fig S2 [file JCMM-24-6500-s002.tif]

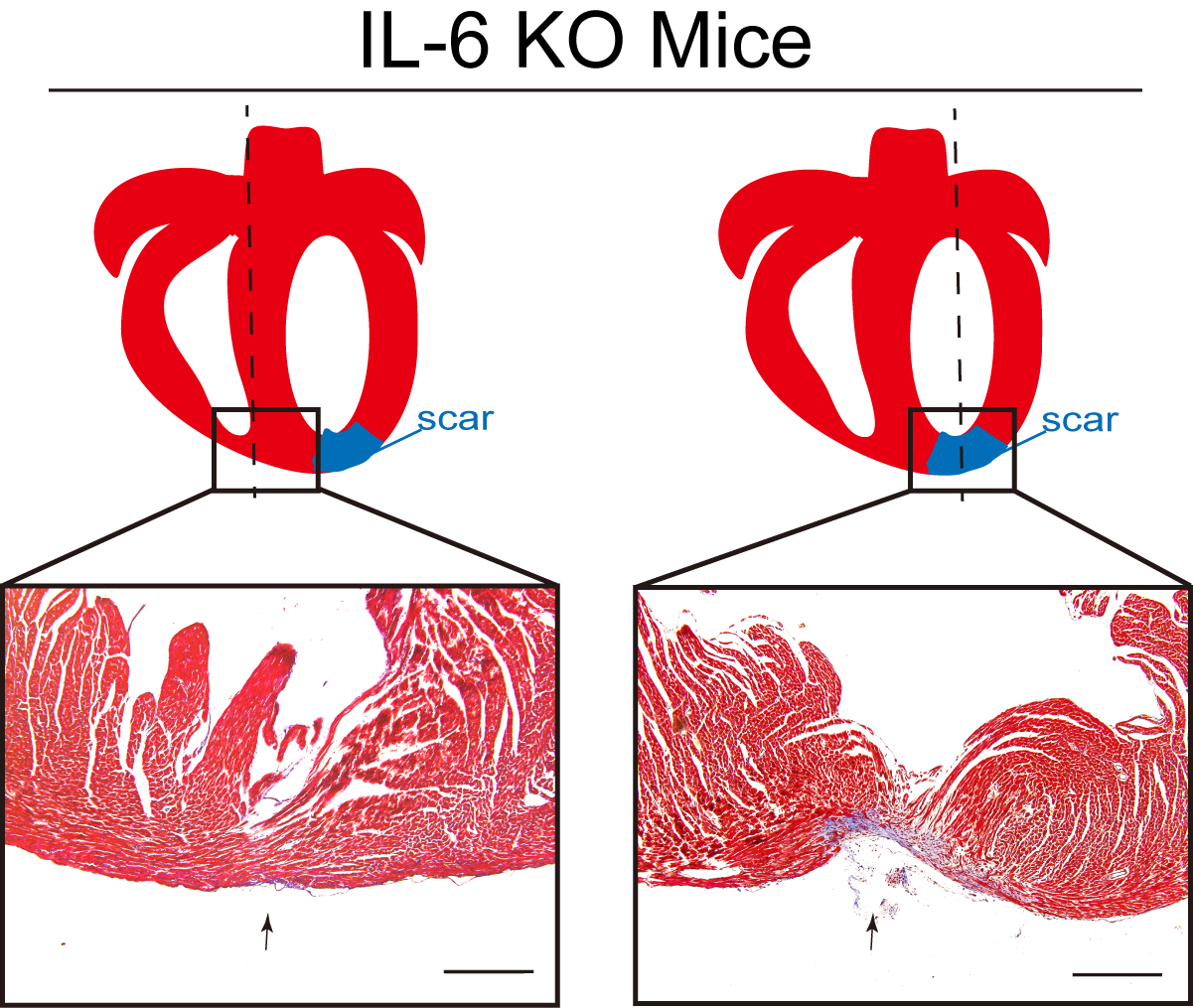

Supplement: Supplementary file 3 — Fig S3 [file JCMM-24-6500-s003.tif]
