# Supplementary material for: Achieving stable myocardial regeneration after apical resection in neonatal mice
Source: J Cell Mol Med. 2020 Apr 28;24(11):6500–4. doi: 10.1111/jcmm.15223 (PMC7294131; doi:10.1111/jcmm.15223)
Supplement: Supplementary file 4 — Supplementary Material [file JCMM-24-6500-s004.docx]

**Supplemental Figure legends**

**Figure S1** Different cutting angles of an apical resection (AR) operation

(A) Ideal cutting angles of AR operation were 60-80°to the long axis (LA) of the heart. (B-C) Cutting angles smaller than 60° or greater than 80° were insufficient to expose the ventricular chamber. (D) Cutting angles greater than 90° inappropriately exposed the right ventricular chamber instead of the left one.

**Figure S2** Images of different cutting times

**Figure S3** Masson’s staining of different regions of *IL-6 KO* mouse heart after AR
